# Biomechanics of World-Class Men and Women Hurdlers

**DOI:** 10.3389/fspor.2021.704308

**Published:** 2021-07-08

**Authors:** Brian Hanley, Josh Walker, Giorgos P. Paradisis, Stéphane Merlino, Athanassios Bissas

**Affiliations:** ^1^Carnegie School of Sport, Leeds Beckett University, Leeds, United Kingdom; ^2^Athletics Sector, School of Physical Education and Sport Science, National and Kapodistrian University of Athens, Athens, Greece; ^3^International Relations and Development Department, World Athletics, Monte Carlo, Monaco; ^4^Athletics Biomechanics, Leeds, United Kingdom; ^5^School of Sport and Exercise, University of Gloucestershire, Gloucester, United Kingdom

**Keywords:** coaching, elite-standard athletes, kinematics, speed, track and field

## Abstract

The sprint hurdle events require athletes to cross ten hurdles between the start and finish line. The height of the hurdles, and the distances between them, differ for men and women, possibly resulting in technical differences. The aim of this study was to provide a kinematic comparison of in-competition hurdle technique for world-class men and women hurdlers. Video data were collected for the 16 finalists in the 100 m and 110 m hurdles events at the 2017 IAAF World Championships using four high-speed cameras (150 Hz), focusing on the sixth hurdle for the men and fifth for the women. Center of mass (CM) position, joint angles, step lengths and clearance times were compared between sexes at key events before, during and after hurdle clearance. The hurdle height was ~7% higher for men when calculated as a proportion of stature (*p* < 0.001). This discrepancy in relative hurdle height provided women with a kinematic and mechanical advantage over men as they took off farther from the hurdle (relative to hurdle height) (*p* < 0.001), leading to a lower and more efficient flight parabola. Women were also able to maintain longer relative step lengths after hurdle clearance and showed minimal vertical oscillation of the CM in the stance phases before and after the hurdle compared with men. The lower relative hurdle heights in the women's event provide a less demanding task, and thus these findings present preliminary evidence to those coaches who advocate revising the women's hurdle heights in competition.

## Introduction

The hurdle events are part of the track and field athletics program at the Olympic Games and all other outdoor major championships. The athletes must cross ten obstacles at set distances, making the event highly technical as the hurdlers try to minimize contact with each barrier while maintaining forward velocity. The sprint hurdle races are held over 100 m for women and 110 m for men, where the women's hurdles are 0.838 m (2'9”) high and the men's hurdles are 1.067 m (3'6”) high. The distance between hurdles in the women's race is 8.50 m, with a 13.00 m approach run and a 10.50 m run-out to the finish; in the men's race the distance between hurdles is 9.14 m, with a 13.72 m approach run and a 14.02 m run-out (World Athletics, 2019). The closeness of the first hurdle to the start line results in a different start technique from that used by sprinters (Bezodis et al., 2019) and, although it is the fastest athlete over the total race distance who wins, the height and distance between the hurdles has a profound effect on the running speeds achieved, with hurdlers not reaching peak speeds until the run-out (Graubner and Nixdorf, 2011). This restriction on speed is not only apparent when crossing the hurdle itself but when recovering speed after clearing it, and in preparing for the next hurdle (McDonald and Dapena, 1991). An analysis of the steps taken after landing from the hurdle clearance would therefore assist coaches to develop a better understanding of the interaction between hurdle clearance and its effect on subsequent steps.

**Figure 1:**
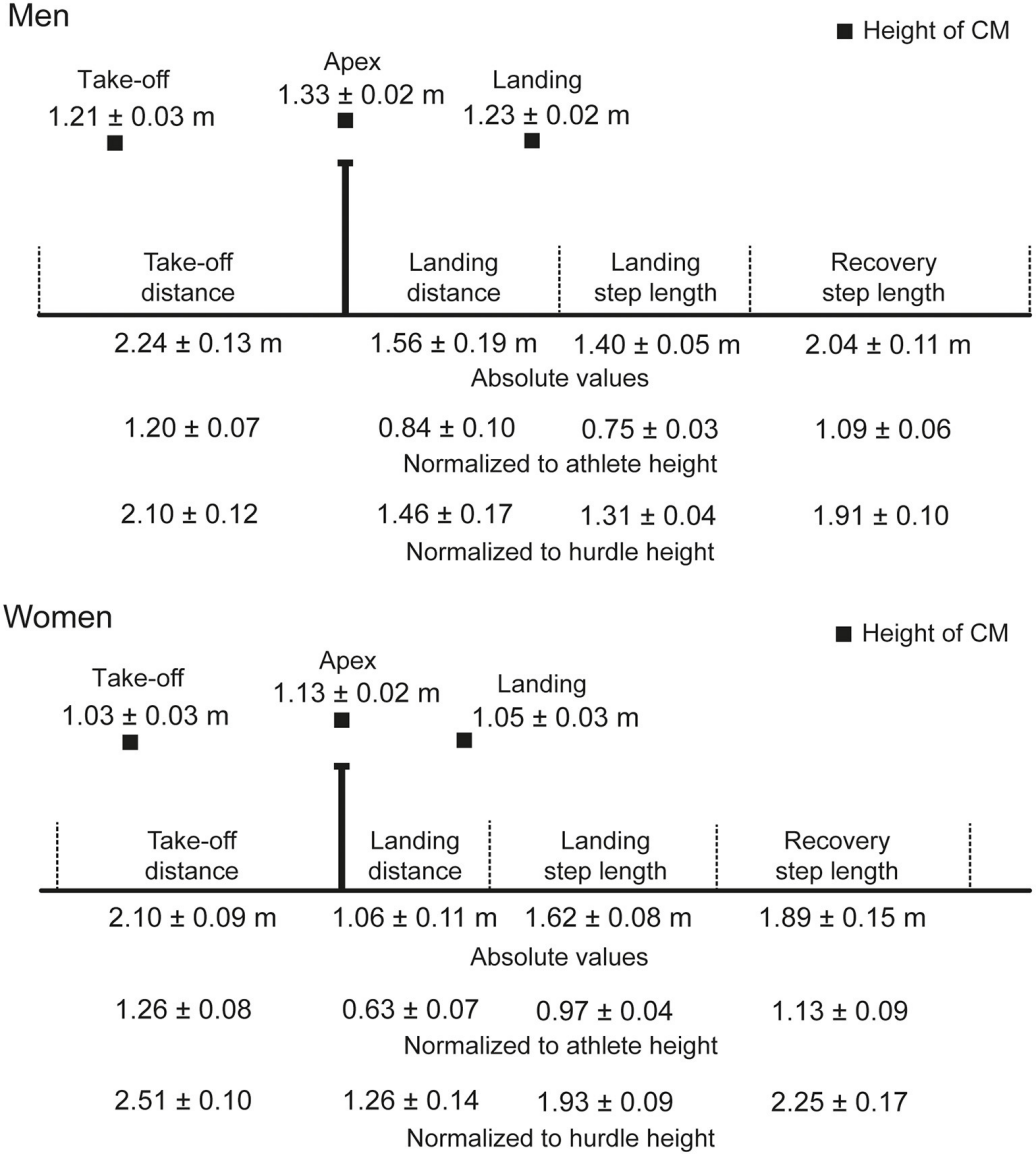
Visual representation of the take-off and landing distances, landing and recovery step lengths, and the height of the CM at take-off and landing. The barrier is shown as the athletes would approach it running from left to right. The diagram is approximately to scale, with separate diagrams for men and women. The mean values (± SD) are shown as absolute values and, for the distances, as normalized to athlete and hurdle height.

After landing from the hurdle step, athletes take three steps between the hurdles, which comprise the landing, recovery and preparatory steps (McDonald and Dapena, 1991; González-Frutos et al., 2019) (Figure 1). Given that these steps' lengths are limited, coaches have recommended approximate lengths for these distances (and the hurdle step) so that athletes maintain forward velocity. For example, in his “model technique” for the men's 110 m hurdles, Tidow (1991) advocated a hurdle step length of ~3.50 m for men, consisting of 2.10–2.20 m before the hurdle and 1.30–1.40 m after it. The ratio used is therefore approximately 60:40 (Čoh et al., 2020), and Salo et al. (1997) similarly recommended a long take-off distance as it allows for a lower flight parabola and better maintenance of horizontal velocity. Because velocity cannot be recovered until ground contact, Salo et al. (1997) stated that the relatively short landing distance after the hurdle helps the athlete to bring the lead leg (the first leg over the hurdle) down more rapidly, which could affect the joint angles at the knee and ankle. This landing component of hurdle step technique is important to develop correctly (Čoh, 2003) as the hurdler must avoid the large horizontal braking impulses that accrue from the foot landing too far in front of the whole body center of mass (CM) (McLean, 1994). Regarding step lengths between the women's hurdles, Hücklekemkes (1990) suggested distances of 1.65, 1.95 and 1.85 m for the landing, recovery and preparatory steps, respectively, based on coaching expertise. In an analysis of hurdlers using three-dimensional (3D) videography (50 Hz), McDonald and Dapena (1991) found that the absolute landing and recovery step lengths differed little between national-standard men and women (by 0.03 and 0.08 m, respectively), but whether these similar values are found in world-class athletes has not been studied. Furthermore, a 3D study using a higher sampling rate would allow for greater precision in identifying specific events such as take-off and touchdown, which can give a more accurate assessment of kinematic and spatiotemporal aspects of hurdling performance, such as step lengths and clearance time. Given that much of the previous recommendations on hurdling have been based on coaches' observations, small sample sizes, or non-elite athletes, a novel analysis of World Championship finalists will provide robust evidence regarding spatiotemporal and kinematic recommendations for elite-standard men's and women's hurdling.

Although coaches have suggested approximate step measurements for athletes to take (e.g., Hücklekemkes, 1990), the length of any steps can depend greatly on athlete stature and thus their step length. Being taller can help clear the hurdle but also hinder a naturally long step length in the three steps between hurdles. It is noteworthy that although the men's hurdle is 27% higher than the women's, the distance between hurdles is only 8% longer for men, and previous case study research has suggested that crossing the barriers is less disruptive to horizontal velocity in the women's event (Čoh, 2003; Čoh et al., 2019). Indeed, coaching literature has previously suggested that, because the men's hurdle is much higher as a proportion of their mean stature, the women's hurdles are too low for modern athletes (Etcheverry, 1993; Stein, 2000); however, there is nonetheless coaching evidence from individual athletes that better hurdling technique (seen in faster clearance times) can differentiate race performance in world-class women hurdlers (Bedini, 2016). Given the differences between men's and women's hurdling in terms of hurdle heights and positioning, a novel study on world-class athletes analyzed in the ecological setting of a major championship final will aid coaches' understanding of key elements of sprint hurdling and any sex-based differences that should be considered in practical terms, including whether there should be an increase in women' hurdle height. The aim of this observational study was to analyze spatiotemporal factors, comprising CM position before, over and after the hurdle, clearance times, step lengths and knee and ankle joint angles, in world-class men's and women's hurdling.

## Materials and Methods

### Research Approval

Data were collected as part of the London 2017 World Championships Biomechanics Research Project (Pollitt et al., 2018a,b). The use of those data for this study was approved by the IAAF (since renamed World Athletics), who own and control the data, and locally the study was reviewed and approved by Carnegie School of Sport Research Ethics Committee. The participants provided their written informed consent to participate in this study. The study was conducted in accordance with the recognized ethical standards of the Declaration of Helsinki.

### Participants

The eight finalists from the men's 110 m hurdles (age: 27 ± 3; stature: 1.87 ± 0.05 m) and the eight finalists from the women's 100 m hurdles (age: 27 ± 3; stature: 1.68 ± 0.04 m) were analyzed in this study. Athletes' dates of birth and finishing times were obtained from the open-access World Athletics website (World Athletics, 2021) for competitors in both races, whereas their statures were obtained from Matthews (2017).

### Data Collection

All data were collected using four Sony PXW-FS7 high-speed cameras (150 Hz; shutter speed: 1/1250 s; ISO: 2000-4000; FHD: 1920 × 1080 px). Cameras were stationary and positioned along the home straight to focus on the sixth and fifth hurdle for the men's and women's event, respectively. These hurdles were analyzed because the allocated camera positions necessitated the analysis of the mid-section of the track (a hurdle position with 50.58 m and 53.00 m remaining for men and women, respectively). A calibration procedure was carried out before and after each event using a rigid cuboid calibration frame (3.044 m^3^) that comprised 24 control points. The frame was positioned in six specific, predefined locations along and across the track to ensure an accurate definition of a volume covering the area around the hurdle for all eight lanes. This approach produced a large number of non-coplanar control points per calibrated volume and facilitated the construction of bi-lane local coordinate systems, which were then combined into a global coordinate system.

### Data Analysis

The collected video files were imported into SIMI Motion (version 9.2.2, Simi Reality Motion Systems GmbH, Germany) and manually digitized by a single experienced operator to obtain whole-body spatiotemporal and kinematic data. An event synchronization technique (synchronization of four critical instants: take-off foot initial contact, take-off foot toe-off, landing foot initial contact and landing foot toe-off) was applied to synchronize the two-dimensional coordinates from each camera. Each file was first digitized frame-by-frame and, upon completion, adjustments were made using the points-over-frame method (Bahamonde and Stevens, 2006). The digitizing process was centered upon critical events (e.g., touchdown, take-off), and identified 17 key anatomical locations (head, shoulder, elbow, wrist, metacarpophalangeal, hip, knee, ankle, and metatarsophalangeal joint centers). The reliability of the digitizing process conducted has been documented previously (Bezodis et al., 2019). The Direct Linear Transformation (DLT) algorithm (Abdel-Aziz et al., 2015) was used to reconstruct the 3D coordinates of each anatomical location from individual camera's x- and y-image coordinates. de Leva's (1996) body segment parameter models were used to obtain data for the CM.

Several spatiotemporal and kinematic variables were obtained from the digitized files. Before the hurdle, take-off distance was defined as the horizontal distance from the metatarsophalangeal joint (representing the part of the foot nearest the ground) at take-off to the base of the hurdle. Distance and height of the CM were defined as the horizontal and vertical positions of the CM relative to the metatarsophalangeal joint, respectively, for touchdown and take-off before and after the hurdle clearance. After hurdle clearance, landing distance was defined as the horizontal distance from the metatarsophalangeal joint to the hurdle. Landing step length was defined as the horizontal distance covered (from touchdown to contralateral touchdown) by the first step after hurdle clearance, and recovery step length was similarly defined for the following step. These variables were computed as absolute values, relative to each athlete's stature and to the height of the hurdle. The knee angle was a sagittal plane angle defined by the three points of the hip, knee and ankle joint centers. The ankle joint was a sagittal plane angle defined by the three points of the knee, ankle and metatarsophalangeal joint centers.

### Statistics

Results are reported as individual values or as means ± one standard deviation (SD). All statistical analyses were carried out using SPSS Statistics 26 (IBM SPSS, Inc., Chicago, IL). Independent samples *t*-tests were used to compare differences between men and women athletes for all variables; significance was set at *p* < 0.05 (Field, 2009). Additionally, Cohen's *d* (Cohen, 1988) was used as an effect size to determine the magnitude of the differences between groups with interpretation thresholds of 0.2 (small), 0.5 (medium), 0.8 (large), 1.2 (very large), and 2.0 (huge).

## Results

**Figure 2:**
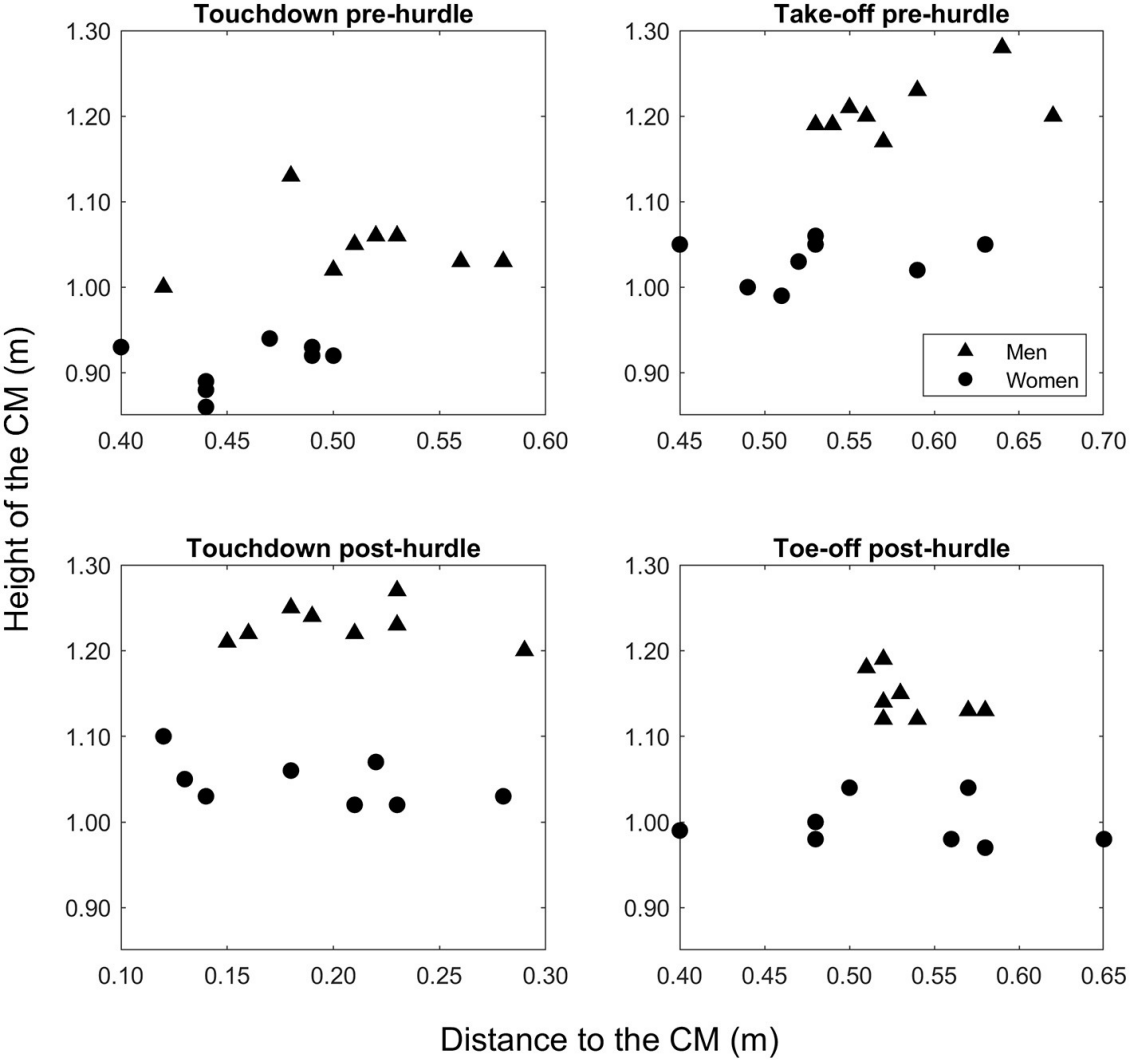
Absolute values for the height of the CM for each man and woman at touchdown and toe-off in the stance phases pre- and post-hurdle.

**Figure 3:**
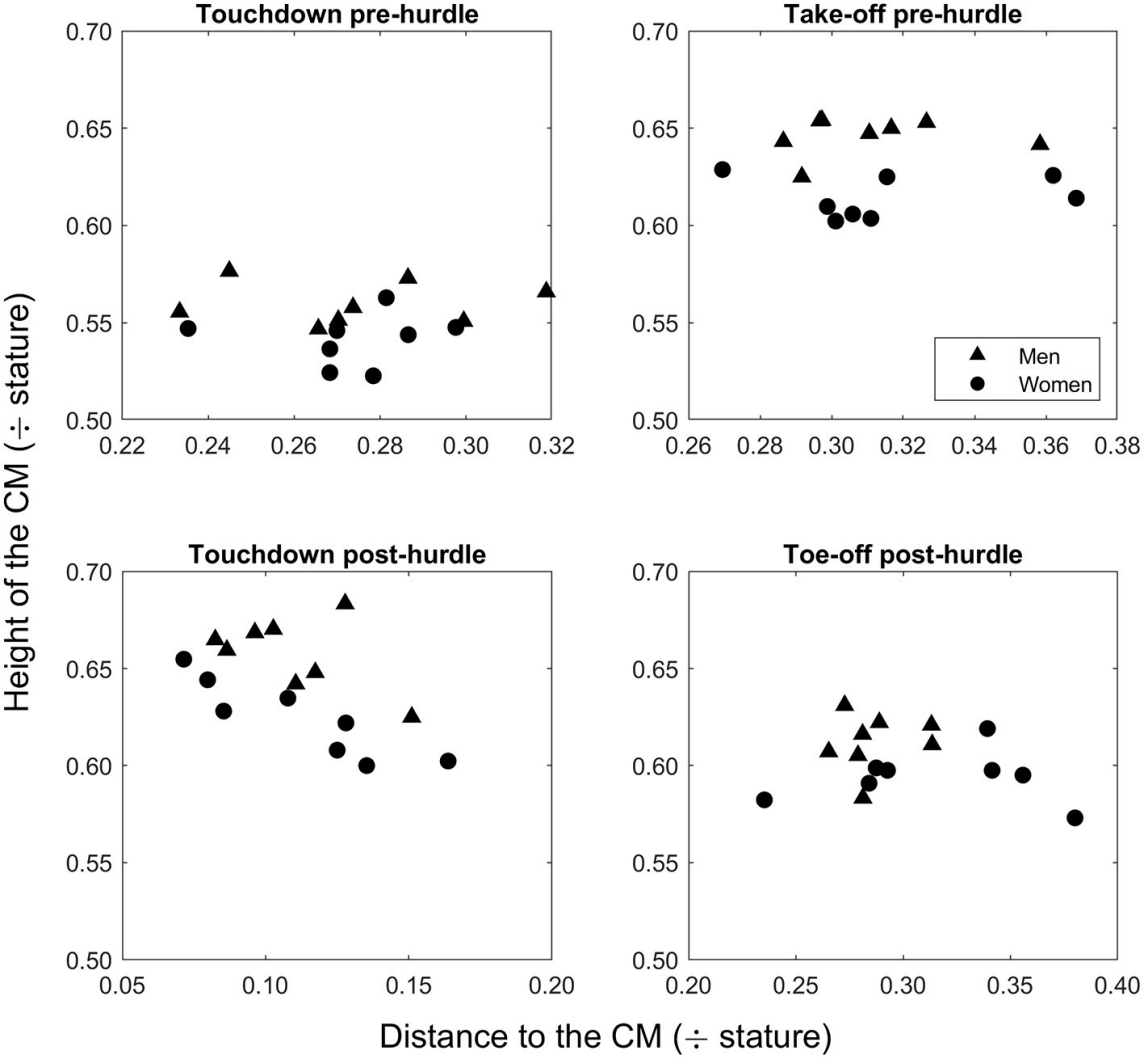
Stature-normalized values for the height of the CM for each man and woman at touchdown and toe-off in the stance phases pre- and post-hurdle.

**Figure 4:**
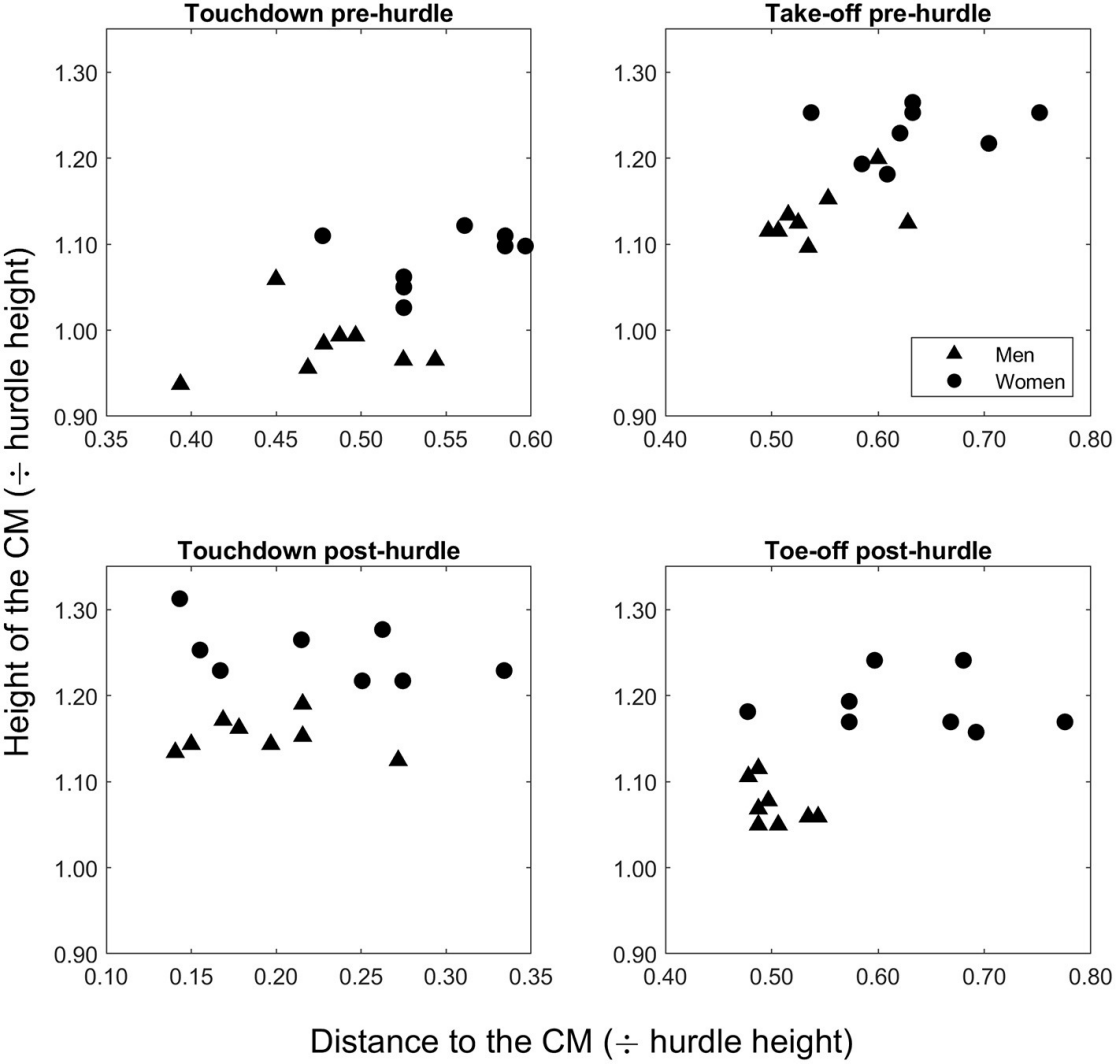
Hurdle height-normalized values for the height of the CM for each man and woman at touchdown and toe-off in the stance phases pre- and post-hurdle.

The mean finishing time in the men's race was 13.27 s (± 0.12), whereas it was 12.76 s (± 0.14) in the women's race. The men were taller than the women (*p* < 0.001, *d* = 3.95), and the barrier height for men was greater when normalized to stature (57.1 ± 1.6%) than the women's hurdle height was for them (49.9% ± 1.3) (*p* < 0.001, *d* = 4.89). Mean hurdle step length was 3.80 m (± 0.13) in the men's race and 3.16 m (± 0.11) in the women's event. The clearance time for men (0.33 ± 0.02 s) was longer than for women (0.28 ± 0.02 s) (*p* < 0.001, *d* = 3.00). In the men's event, the take-off distance was ~59% of total hurdle step length, whereas for women it was 66% (Figure 1). In absolute terms, the men's take-off distance was longer by 0.14 m (*p* = 0.032, *d* = 1.19), but the women's take-off distance was longer when normalized for hurdle height (*p* < 0.001, *d* = 3.64). The height of the CM at take-off was higher in men both in absolute terms (*p* < 0.001, *d* = 5.92) (Figure 2) and normalized to stature (men: 0.65 ± 0.01; women: 0.61 ± 0.01; *p* < 0.001, *d* = 2.81) (Figure 3), but was higher in women when normalized to hurdle height (men: 1.13 ± 0.03; women: 1.23 ± 0.03; *p* < 0.001, *d* = 3.18) (Figure 4). The height of the CM increased in men by 0.16 m (± 0.03) from touchdown to toe-off during the stance phase before the hurdle, and in women by 0.12 m (± 0.01) (Figure 2). Men's clearance heights (flight parabola apex) were higher by 0.20 m (*p* < 0.001, *d* = 11.26) (Figure 1), which was also higher normalized to stature (men: 0.71 ± 0.02; women: 0.67 ± 0.02; *p* = 0.001, *d* = 2.08), but lower relative to hurdle height (men: 1.25 ± 0.01; women: 1.35 ± 0.02; *p* < 0.001, *d* = 4.94).

Men's landing distances after clearing the hurdle were longer than women's as absolute values and when normalized to stature and hurdle height (Figure 1) (all *p* ≤ 0.022, *d* ≥ 1.31). The height of the CM was higher in men at touchdown by 0.18 m (Figure 2) (*p* < 0.001, *d* = 7.14), which was also higher than in women when normalized to stature (men: 0.66 ± 0.02; women: 0.62 ± 0.02; *p* = 0.004, *d* = 2.00) (Figure 3) but lower when normalized to hurdle height (men: 1.15 ± 0.02; women: 1.25 ± 0.03; *p* < 0.001, *d* = 3.60) (Figure 4). The height of the CM decreased from touchdown to toe-off during the landing contact phase in men by 0.09 m (± 0.01) and in women by 0.05 m (± 0.02) (Figure 2). The knee angle at touchdown was 166° (± 10) in men, greater than that found in women (156° ± 9) (*p* = 0.037, *d* = 1.15); there was no difference in ankle angle (men: 130° ± 11; women: 126° ± 7).

The landing step was the only step longer in women when expressed in absolute values (*p* < 0.001, *d* = 3.51) (Figure 1). Men had longer recovery steps in absolute values (*p* = 0.032, *d* = 1.19) but these were longer in women when normalized to hurdle height (*p* < 0.001, *d* = 2.41), but not stature. Although not directly measured, knowing the set distance between hurdles and these other step lengths indicate that the men's preparatory step length was approximately 1.90 m, whereas the women's was 1.83 m.

## Discussion

The aim of this observational study was to analyze spatiotemporal factors, comprising CM position before, over and after the hurdle, clearance times, step lengths and knee and ankle joint angles, in world-class men's and women's hurdling. Amongst the finalists in the 2017 World Championships, the men's hurdle height corresponded to 57.1% of their mean stature, whereas the women's hurdle corresponded to only 49.9% of theirs. As a result, spatiotemporal aspects of the hurdle step were different between men and women, even when taking stature into account. This included the height of the CM during the stance phase during both the preparation for take-off before the hurdle, its flight apex, and when landing after it. When normalized to hurdle height, men's CMs travel closer to the top of the hurdle whereas women are relatively higher, meaning that women have a larger margin for error during clearance. Indeed, women deliberately trying to cross the hurdle lower could require more effort and loss of speed. By contrast, men adopt a technique that minimizes their already high parabola. For example, the men had higher CM positions relative to stature at both initial contact and toe-off before and after clearance, meaning that women do not need to raise their CM as much to clear the hurdle, and the easier maintenance of horizontal speed allows women to have relatively longer step lengths during both landing and recovery steps. This difference becomes more apparent when comparing the CM heights expressed relative to hurdle height, where women's CM values are much higher than the hurdle height compared with men. The ratio of take-off to landing distance during the hurdle step for men was 59:41, practically identical to what was recommended by Tidow (1991), whereas for women it was 66:34. Given that longer take-off distances allow for lower flight parabolas and better maintenance of horizontal velocity (Salo et al., 1997), it is clear that a lower relative hurdle height means that women require less effort to project the body upward and maintaining velocity is less disrupted (Čoh, 2003; Čoh et al., 2019), resulting in an overall less demanding task with more horizontal running and less demand for applying ground reaction forces. Indeed, the technical difference between national-standard and elite-standard women hurdlers over 60 m is less than in the same groups amongst men (González-Frutos et al., 2019). In coaching literature, Etcheverry (1993) and Stein (2000) have both advocated an increase in women's hurdle height to 0.914 m (3'0”). Such an increase in height would represent 54.5% of the mean stature of the women analyzed in this study, still lower than the men's relative height but presenting a more similar challenge.

Clearing the hurdles is easier if the take-off position of the CM is higher, and men's relatively higher take-off and touchdown positions during the hurdle step require a greater raise of their CM during the take-off stance phase and a greater drop in CM position during the landing stance phase, respectively. Overall, this means that men's CM positions fluctuate more during the stance phases at either end of the hurdle step; however, when normalizing the same variables for hurdle height there is no difference between men and women. It seems that when absolute and stature-normalized values are used, men's CM positions fluctuate more vertically than women to complete the task, with corresponding effects on step lengths and clearance time. When these fluctuations are compared based on hurdle height, the two groups appear equal because men sacrifice all other mechanics to adjust to the hurdle's height, and the height of the women's hurdle does not need specific tuning but instead allows a smoother flow between both initial contact and toe-off both before and after the hurdle. Men's higher position at landing was achieved by adopting a more extended knee at touchdown, and the drop in the CM position to toe-off in this stance phase requires considerable leg strength to avoid too great a lowering of the CM before the landing step. Given the landing leg's important role in reducing landing distance to avoid unnecessarily large braking forces and to recover forward velocity (McLean, 1994; Salo et al., 1997), coaches are advised to develop lower leg strength in supporting the body on landing in all hurdlers, but possibly more so for men, via an additional focus on eccentric knee extensor strength to allow for higher knee stiffness and a more extended knee at landing.

The height of the hurdle does not just affect how men and women cross it differently, but also has a profound effect on the sex-based differences in step lengths between the hurdles. All distances (apart from landing distance, which is a more passive distance, i.e., landing from the hurdle) are longer for women in relative terms, with the landing step length longer in women even when expressed in absolute terms. This means (in particular when normalizing for stature) a more distance-optimal forward movement for women, in that they cover more distance per step for their stature compared with men. This is not the case when considering, for example, the 100 m finals from the same 2017 World Championships where the mean stature-normalized step length for men was 1.21, whereas for women it was 1.19 (Bissas et al., 2018a,b). Accordingly, during late stance propulsion in the pre-hurdle contact phase, ending with take-off, women propel their bodies forward more with respect to their height; it is possible to therefore assume that women are more efficient with regard to force production and energy consumption, and could explain why women hurdlers are not trained to produce as much horizontal force as women sprinters (Stavridis et al., 2019). Indeed, if women traveled the same relative distance as men for the landing and recovery step lengths, this would have meant traveling on average (using the group mean) 3.08 m for these two steps compared with the measured 3.51 m. As this would have implications in all subsequent steps before the next hurdle, the current hurdle height therefore provides women with a “kinematic” and perhaps mechanical advantage (which might be measured in future studies using kinetic analyses). Therefore, the hurdle height not only affects the clearance phase but also the steps between the hurdles and energy requirements, and raising the women's hurdle height would have a consequent effect on these elements of the race. There is thus ecological evidence to support coaches' historical recommendations that the women's hurdle height be raised to 0.914 m. However, it should be noted that it is not essential that men's and women's events are “equivalent”, and that there would be considerable effects on the training methods and technical requirements of women hurdlers, including those who compete in the heptathlon, where the 100 m hurdles is the first of the seven events. Nonetheless, the findings of the present study provide a scientific basis for revising the hurdle heights currently used to increase similarity between men and women's hurdling events. Few studies have been conducted in laboratory conditions to measure relevant kinetic variables such as impulse and braking forces, but future studies of this nature could inform coaching practice in concert with the kinematic and spatiotemporal findings from this study of the world's best hurdlers.

The main strength of this novel study is that the data are of world-class hurdlers competing in World Championship finals, and therefore the research has high ecological validity. Additionally, the competitors analyzed were the largest group of elite-standard hurdlers ever studied, and mean that the results can be used by coaches as a model of excellence. For example, we found the absolute distances for the hurdle step, landing step, recovery step and preparatory step (estimated) to be 3.80, 1.40, 2.04 and 1.90 m for men, and 3.16, 1.62, 1.89 and 1.83 m for women, similar to those value predicted by coaches (e.g., Hücklekemkes, 1990). However, the nature of the event's structure means that the sample was limited to eight athletes in each race, and recording performances in competition was constrained to analyzing the kinematics of one mid-race hurdle clearance only, meaning that our analyses will apply mostly to those hurdles where running speed is relatively constant (i.e., from the third hurdle to the ninth) (Pollitt et al., 2018a,b). The clearance of the first hurdle, in particular, could be quite different given slower running speeds, with fatigue also potentially affecting clearance of the last hurdle. Additionally, the clearance of the last hurdle could differ from the others as the athletes no longer need concern themselves with preparing for a step pattern that must accommodate the approach to a hurdle. Nonetheless, by focusing all recording on a single hurdle clearance meant that the four cameras used for 3D analysis provided extensive coverage of the hurdling motion and successive steps, and by using high-definition high-speed cameras, it was possible to obtain a precision of analysis not used before in outdoor competition (e.g., McDonald and Dapena, 1991). Future biomechanical studies at world-class competitions that focus on other sections of the race, such as hurdles earlier or later in the race, would complement these findings and provide more information to coaches on key factors in hurdling success.

## Conclusions

In summary, this was the first study to analyze hurdling kinematics in a group of world-class athletes within the highly ecological setting of a World Championships. The men's hurdle was about 7% higher relative to their stature than the women's hurdle was for them, resulting in a more energy-costly vertical displacement of the CM in men not only over the hurdle but also during the take-off and landing stance phases. The relatively higher hurdle of the men's event also required a more extended knee upon landing, and emphasized the landing leg's role in supporting the body effectively regarding moving into the subsequent landing step. Women were also able to take off farther from the hurdle in relative terms, meaning a less demanding task and affecting the step lengths achieved between the hurdles. Overall, the lower hurdle heights for women, relative to stature, provide them with a kinematic and potentially mechanical advantage over the men.

## Data Availability Statement

The raw data supporting the conclusions of this article will be made available by the authors, without undue reservation.

## Ethics Statement

The studies involving human participants were reviewed and approved by Carnegie School of Sport Research Ethics Committee, Leeds Beckett University. The patients/participants provided their written informed consent to participate in this study.

## Author Contributions

BH, JW, and AB performed data collection. JW, GP and AB processed the data. BH and JW created the figures. All authors conceptualized and designed the study, wrote the manuscript, interpreted the results of the research, edited, critically revised, and approved the final version for submission.

## Conflict of Interest

The authors declare that the research was conducted in the absence of any commercial or financial relationships that could be construed as a potential conflict of interest.
